# Supplementary material for: ActinoMation: A literate programming approach for medium-throughput robotic conjugation of Streptomyces spp
Source: Synth Syst Biotechnol. 2025 Mar 11;10(2):667–76. doi: 10.1016/j.synbio.2025.03.005 (PMC11999424; doi:10.1016/j.synbio.2025.03.005)
Supplement: Multimedia component 1 [file mmc1.pdf]

SUPPORTING INFORMATION

ActinoMation: a literate programming approach for medium-throughput robotic conjugation of *Streptomyces* spp.

Tenna Alexiadis Møller[1], Thom Booth[1], Simon Shaw[1], Vilhelm Krarup Møller[2], Rasmus Frandsen[2], Tilmann Weber[1]

[1]The Novo Nordisk Foundation Center for Biosustainability, Technical University of Denmark, Kgs. Lyngby, Denmark.

[2]DTU Bioengineering, Technical University of Denmark, Kgs. Lyngby, Denmark.

Contents

SUPPLEMENTAL FIGURES 2

Figure S1: Example Layouts from ActinoMation. 2

Figure S2: Dependency tree for the conjugation and transformation workflow notebooks. 3

Figure S3: Agarose gel of colony PCR results. 4

SUPPLEMENTAL TABLES 5

Table S1: Primer design used for colony PCR. 5

Table S2: Plate design for conjugation on the Opentrons 2. 6

Table S3: Exconjugant colonies counted on MS agar plates. 7

Table S4: Conjugation efficiency. 8

Table S5: True conjugation efficiency. 9

SUPPLEMENTAL FIGURES

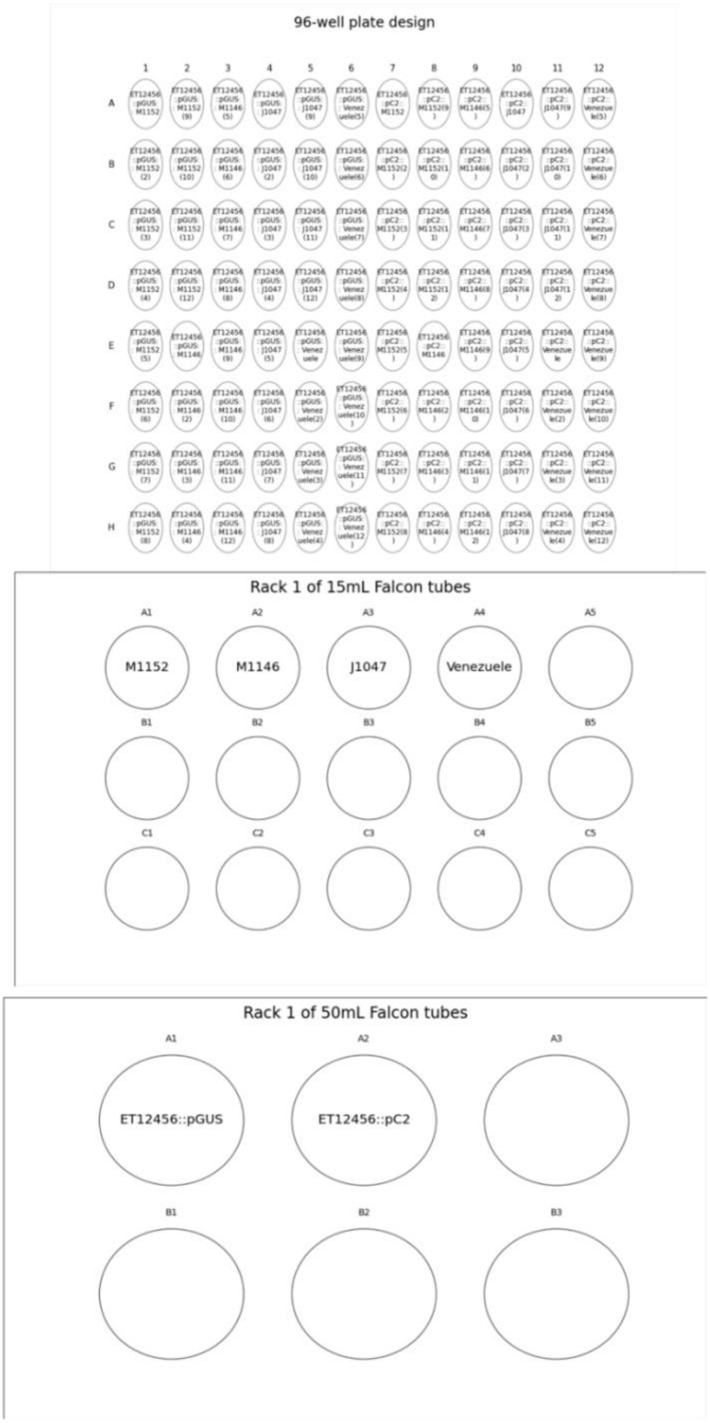

Figure S1: Example Layouts from ActinoMation. An example of the visual representation produced by the ActinoMation Jupyter Notebook of the final plate and rack layout including sample names and combinations.

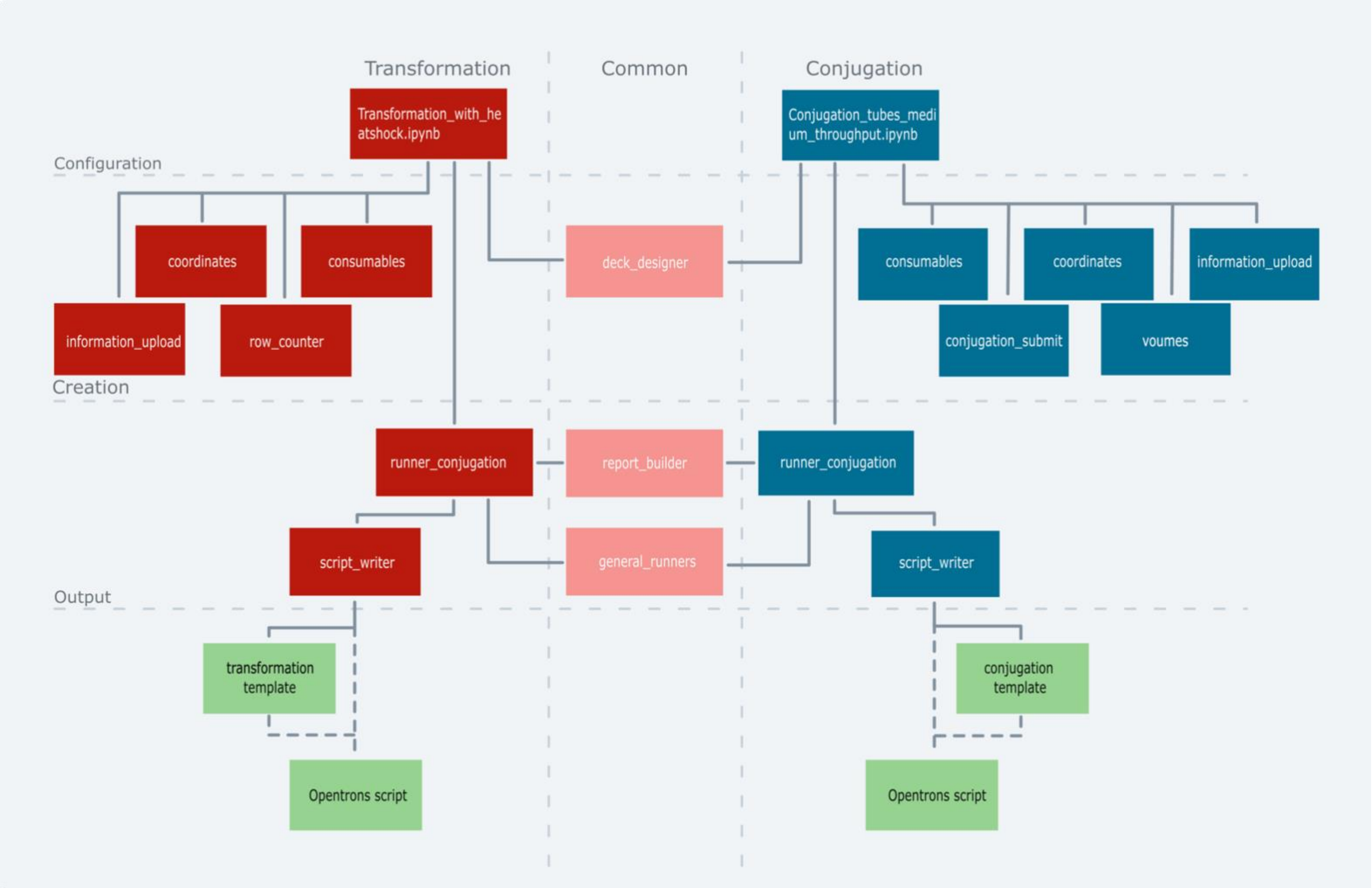

Figure S2: Dependency tree for the conjugation and transformation workflow notebooks. Each square represents a python module containing related functions. The tree is divided into three columns: the left for heat-shock transformation protocol functions, the right for conjugation functions, and the middle for functions shared by both workflows. Horizontally, the tree splits into three sections: "configuration" for user-modifiable protocol functions, "creation" for metadata and Opentrons protocol creation, and "output" for the final product template. The two top squares are Jupyter Notebook files.

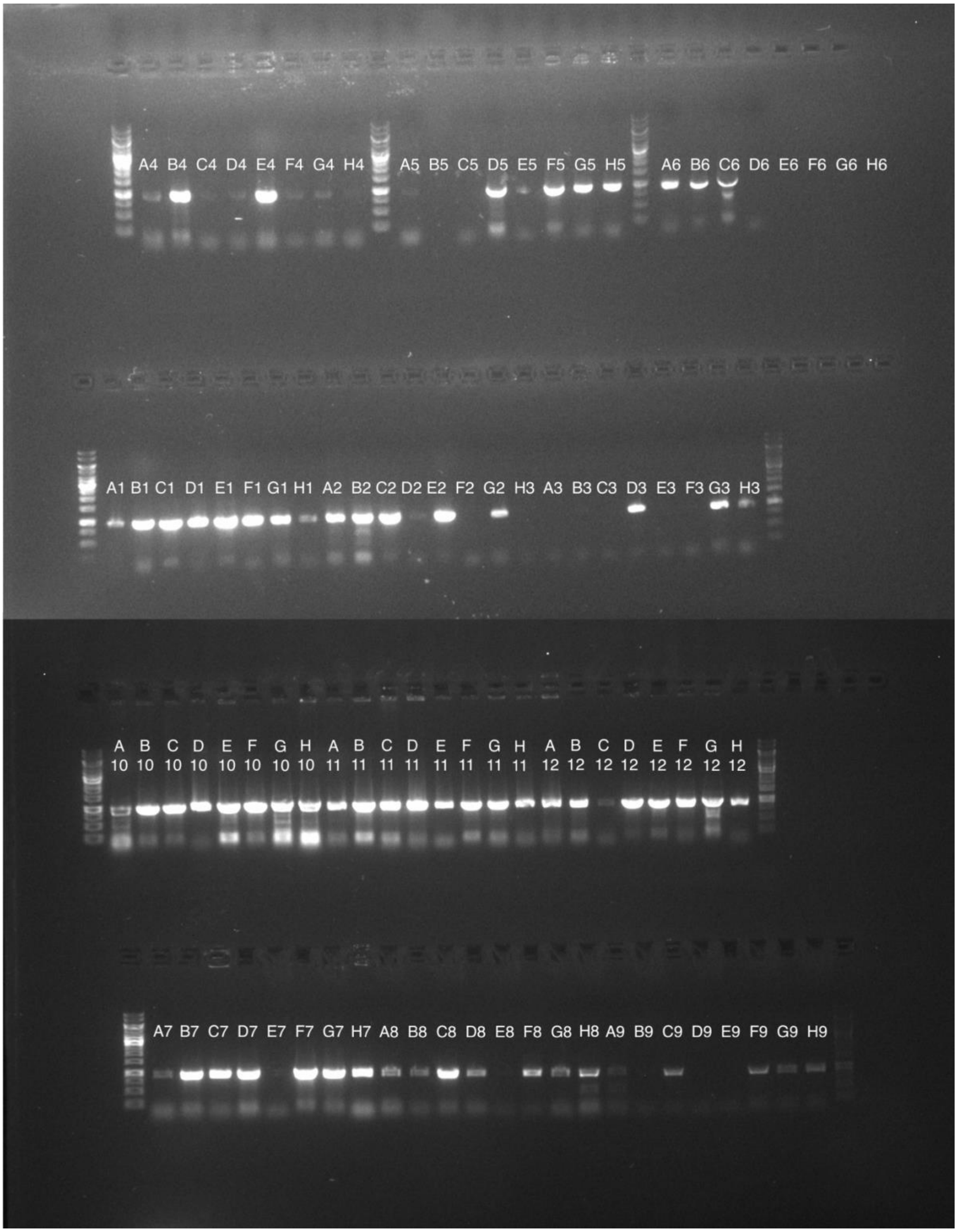

Figure S3: Agarose gel of colony PCR results. Exconjugation for pSETGUS and pIJ12551 with M1152, M1146, J1047 and DSM40230. Letters and numbers correspond with table S2.

SUPPLEMENTAL TABLES

Primer name	Sequence	Concentration	Purpose
**pRM4e-apmR-LF**	5’-AGCAGCCACTGGTAAC-3’	10 mM	Forward Primer
**pRM4e-apmR-LR**	5’-ATGCAGCGTCGTGTT-3’	10 mM	Reverse Primer

Table S1: Primer design used for colony PCR. Table for the two primers, forward and reverse, used in the colony PCR. Primers are custom oligos provided by IDT (Integrated DNA Technologies; IA, USA).

	1	2	3	4	5	6	7	8	9	10	11	12
A	ET12456::p<br>SETGUS::<br>M1152	ET12456::p<br>SETGUS::<br>M1152	ET12456::p<br>SETGUS::<br>M1146	ET12456::p<br>SETGUS::<br>J1047	ET12456::p<br>SETGUS::<br>J1047	ET12456::p<br>SETGUS::<br>DSM40230	ET12456::p<br>IJ12551::<br>M1152	ET12456::p<br>IJ12551::<br>M1152	ET12456::p<br>IJ12551::<br>M1146	ET12456::p<br>IJ12551::<br>J1047	ET12456::p<br>IJ12551::<br>J1047	ET12456::p<br>IJ12551::<br>DSM40230
B	ET12456::p<br>SETGUS::<br>M1152	ET12456::p<br>SETGUS::<br>M1152	ET12456::p<br>SETGUS::<br>M1146	ET12456::p<br>SETGUS::<br>J1047	ET12456::p<br>SETGUS::<br>J1047	ET12456::p<br>SETGUS::<br>DSM40230	ET12456::p<br>IJ12551::<br>M1152	ET12456::p<br>IJ12551::<br>M1152	ET12456::p<br>IJ12551::<br>M1146	ET12456::p<br>IJ12551::<br>J1047	ET12456::p<br>IJ12551::<br>J1047	ET12456::p<br>IJ12551::<br>DSM40230
C	ET12456::p<br>SETGUS::<br>M1152	ET12456::p<br>SETGUS::<br>M1152	ET12456::p<br>SETGUS::<br>M1146	ET12456::p<br>SETGUS::<br>J1047	ET12456::p<br>SETGUS::<br>J1047	ET12456::p<br>SETGUS::<br>DSM40230	ET12456::p<br>IJ12551::<br>M1152	ET12456::p<br>IJ12551::<br>M1152	ET12456::p<br>IJ12551::<br>M1146	ET12456::p<br>IJ12551::<br>J1047	ET12456::p<br>IJ12551::<br>J1047	ET12456::p<br>IJ12551::<br>DSM40230
D	ET12456::p<br>SETGUS::<br>M1152	ET12456::p<br>SETGUS::<br>M1152	ET12456::p<br>SETGUS::<br>M1146	ET12456::p<br>SETGUS::<br>J1047	ET12456::p<br>SETGUS::<br>J1047	ET12456::p<br>SETGUS::<br>DSM40230	ET12456::p<br>IJ12551::<br>M1152	ET12456::p<br>IJ12551::<br>M1152	ET12456::p<br>IJ12551::<br>M1146	ET12456::p<br>IJ12551::<br>J1047	ET12456::p<br>IJ12551::<br>J1047	ET12456::p<br>IJ12551::<br>DSM40230
E	ET12456::p<br>SETGUS::<br>M1152	ET12456::p<br>SETGUS::<br>M1146	ET12456::p<br>SETGUS::<br>M1146	ET12456::p<br>SETGUS::<br>J1047	ET12456::p<br>SETGUS::<br>DSM40230	ET12456::p<br>SETGUS::<br>DSM40230	ET12456::p<br>IJ12551::<br>M1152	ET12456::p<br>IJ12551::<br>M1146	ET12456::p<br>IJ12551::<br>M1146	ET12456::p<br>IJ12551::<br>J1047	ET12456::p<br>IJ12551::<br>DSM40230	ET12456::p<br>IJ12551::<br>DSM40230
F	ET12456::p<br>SETGUS::<br>M1152	ET12456::p<br>SETGUS::<br>M1146	ET12456::p<br>SETGUS::<br>M1146	ET12456::p<br>SETGUS::<br>J1047	ET12456::p<br>SETGUS::<br>DSM40230	ET12456::p<br>SETGUS::<br>DSM40230	ET12456::p<br>IJ12551::<br>M1152	ET12456::p<br>IJ12551::<br>M1146	ET12456::p<br>IJ12551::<br>M1146	ET12456::p<br>IJ12551::<br>J1047	ET12456::p<br>IJ12551::<br>DSM40230	ET12456::p<br>IJ12551::<br>DSM40230
G	ET12456::p<br>SETGUS::<br>M1152	ET12456::p<br>SETGUS::<br>M1146	ET12456::p<br>SETGUS::<br>M1146	ET12456::p<br>SETGUS::<br>J1047	ET12456::p<br>SETGUS::<br>DSM40230	ET12456::p<br>SETGUS::<br>DSM40230	ET12456::p<br>IJ12551::<br>M1152	ET12456::p<br>IJ12551::<br>M1146	ET12456::p<br>IJ12551::<br>M1146	ET12456::p<br>IJ12551::<br>J1047	ET12456::p<br>IJ12551::<br>DSM40230	ET12456::p<br>IJ12551::<br>DSM40230
H	ET12456::p<br>SETGUS::<br>M1152	ET12456::p<br>SETGUS::<br>M1146	ET12456::p<br>SETGUS::<br>M1146	ET12456::p<br>SETGUS::<br>J1047	ET12456::p<br>SETGUS::<br>DSM40230	ET12456::p<br>SETGUS::<br>DSM40230	ET12456::p<br>IJ12551::<br>M1152	ET12456::p<br>IJ12551::<br>M1146	ET12456::p<br>IJ12551::<br>M1146	ET12456::p<br>IJ12551::<br>J1047	ET12456::p<br>IJ12551::<br>DSM40230	ET12456::p<br>IJ12551::<br>DSM40230

Table S3: Plate design for conjugation on the Opentrons 2. Each cell contains the names of the strains usided in combinations of ET12456::pSETGUS or ET12456::PIJ12551 with M1152, M1146, J1047 and DSM40230.

	1	2	3	4	5	6	7	8	9	10	11	12
**A**	>100	>100	>100	0	3	71	>100	>100	>100	63	91	>100
**B**	>100	>100	>100	5	0	58	>100	>100	>100	55	76	>100
**C**	>100	>100	>100	5	0	NA	>100	>100	>100	52	69	>100
**D**	>100	>100	>100	5	8	NA	>100	>100	>100	13	>100	>100
**E**	>100	>100	>100	4	57	NA	>100	>100	>100	>100	>100	>100
**F**	>100	>100	>100	5	113	NA	>100	>100	>100	>100	>100	>100
**G**	>100	>100	>100	3	74	NA	>100	>100	>100	>100	>100	>100
**H**	>100	NA	>100	1	NA	NA	>100	>100	>100	85	4	>100

Table S4: Exconjugant colonies counted on MS agar plates. Each cell corresponds to the conjugation experiment described in Table S1. Cells with NA are shown were data was not counted due to aspiration errors.

	1	2	3	4	5	6	7	8	9	10	11	12
A	0.03333333	0.03333333	0.00296296	0	1.1215E-05	0.04733333	0.03333333	0.03333333	0.00296296	0.00039252	0.00056698	0.06666667
B	0.03333333	0.03333333	0.00296296	1.8692E-05	0	0.03866667	0.03333333	0.03333333	0.00296296	0.00034268	0.00047352	0.06666667
C	0.03333333	0.03333333	0.00296296	1.8692E-05	0	NA	0.03333333	0.03333333	0.00296296	0.00032399	0.00042991	0.06666667
D	0.03333333	0.03333333	0.00296296	1.8692E-05	2.9906E-05	NA	0.03333333	0.03333333	0.00296296	8.0997E-05	0.00062305	0.06666667
E	0.03333333	0.00296296	0.00296296	1.4953E-05	0.038	NA	0.03333333	0.00296296	0.00296296	0.00062305	0.06666667	0.06666667
F	0.03333333	0.00296296	0.00296296	1.8692E-05	0.07533333	NA	0.03333333	0.00296296	0.00296296	0.00062305	0.06666667	0.06666667
G	0.03333333	0.00296296	0.00296296	1.1215E-05	0.04933333	NA	0.03333333	0.00296296	0.00296296	0.00062305	0.06666667	0.06666667
H	0.03333333	NA	0.00296296	3.7383E-06	NA	NA	0.03333333	0.00296296	0.00296296	0.0005296	0.00266667	0.06666667

Table S5: Conjugation efficiency. The conjugation efficiency calculated as the final number of ex-conjugants from Table S2 divided by the initial spore concentration.

	1	2	3	4	5	6	7	8	9	10	11	12
A	0.00305666	0.00305666	0.00134696	0	1.8692E-05	0.04733333	0.00305666	0.00305666	0.00161629	0.00039252	0.00056698	0.06111334
B	0.00305666	0.00305666	0.00134696	3.1153E-05	0	0.03866667	0.00305666	0.00305666	0.00161629	0.00034268	0.00047352	0.06111334
C	0.00305666	0.00305666	0.00134696	3.1153E-05	0	NA	0.00305666	0.00305666	0.00161629	0.00032399	0.00042991	0.06111334
D	0.00305666	0.00305666	0.00134696	3.1153E-05	4.9844E-05	NA	0.00305666	0.00305666	0.00161629	8.0997E-05	0.00062305	0.06111334
E	0.00305666	0.00134696	0.00134696	2.4922E-05	0.038	NA	0.00305666	0.00161629	0.00161629	0.00062305	0.06111334	0.06111334
F	0.00305666	0.00134696	0.00134696	3.1153E-05	0.07533333	NA	0.00305666	0.00161629	0.00161629	0.00062305	0.06111334	0.06111334
G	0.00305666	0.00134696	0.00134696	1.8692E-05	0.04933333	NA	0.00305666	0.00161629	0.00161629	0.00062305	0.06111334	0.06111334
H	0.00305666	NA	0.00134696	6.2305E-06	NA	NA	0.00305666	0.00161629	0.00161629	0.0005296	0.00244454	0.06111334

Table S6: True conjugation efficiency. The efficiency is calculated on data from table S3 and the false positive rates calculated in the main text.
